# Hope on the Horizon: New and Future Therapies for Sickle Cell Disease

**DOI:** 10.3390/jcm12175692

**Published:** 2023-09-01

**Authors:** Fahd A. Kuriri

**Affiliations:** Department of Clinical Laboratory Sciences, College of Applied Medical Sciences, Shaqra University, Shaqra, Riyadh 15572, Saudi Arabia; fkuriri@su.edu.sa; Tel./Fax: +966-116475136

**Keywords:** sickle cell, blood transfusion, stem cell, treatments

## Abstract

This article provides an overview of conventional, new, and future treatment options for sickle cell disease (SCD), a genetic disorder affecting the production of hemoglobin. Current treatments include hydroxyurea, a conventional SCD treatment that increases the levels of fetal hemoglobin, and new treatments such as voxelotor, a recently approved SCD treatment that selectively binds hemoglobin, preventing formation of sickled red blood cells. In addition to discussing the mechanisms of action of current SCD treatments, potential side effects are also discussed, highlighting the need for new treatments that can address the limitations of current treatments and improve the quality of life for people with SCD. Future treatments, such as gene therapy, are also explored as promising treatment options for SCD patients.

## 1. Introduction

Sickle cell disease (SCD) refers to common genetic blood disorders that affect millions of people worldwide causing morbidity and early mortality. In SCD, hemolgobin production is impacted, producing abnormal hemoglobin, known as hemoglobin S, that causes red blood cells (RBCs) to become sickle-shaped and rigid, leading to various health problems including blood flow obstruction, severe pain, and organ damage. Conventional SCD treatments include hydroxyurea (also known as hydroxycarbamide) to help manage pain and prevent complications and blood transfusions to increase the number of healthy RBCs. However, conventional treatments are not always effective and have side effects; subsequently, new treatment strategies are always being explored. In the past six years, three new SCD drugs with different modes of action have been approved by the U.S. Food and Drug Administration (FDA): voxelotor, crizanlizumab, and L-glutamine. Research into curative strategies is ongoing, with many recent studies conducted on the use of stem cell therapy to replace damaged cells and improve the function of the bone marrow. Curative research has also focused recently on gene therapy, a technique intended to correct the genetic defect causing the disorder. This article aims to review conventional SCD treatments alongside new and future treatment options that are providing hope for SCD patients.

## 2. Conventional Treatments

To date, blood transfusions and hydroxyurea remain the main therapeutic strategies for managing SCD. Blood transfusions were first used to reduce recurrent strokes in SCD patients in the 1970s, while hydroxyurea was first approved for use in adults by the FDA in 1998 [1,2].

### 2.1. Hydroxyurea

Hydroxyurea has been used as a first-line management option for sickle cell anemia since the 1990s [3] and has been proven effective for preventing sickle cell disease-related vaso-occlusive crises, which are a common complication of this disorder. Despite its effectiveness, hydroxyurea can cause many side effects including neutropenia, bone marrow suppression, increased hepatic enzymes, anorexia, nausea, and vomiting [2]. Moreover, the potential risk of infertility associated with hydroxyurea induction in individuals with sickle cell disease has gained attention and requires careful consideration [4,5]. It has been suggested that hydroxyurea, despite its benefits in treating the disease, might pose a risk to fertility. This concern arises from the drug’s potential impact on sperm production and reproductive cells. Recent studies, such as the one by L. Joseph et al. have challenged the assumption that hydroxyurea definitively causes infertility [6]. This study suggests that the relationship between hydroxyurea and fertility is more complex than previously thought, with factors such as dose, duration of treatment, and individual patient characteristics potentially influencing the outcome. While the exact mechanisms by which hydroxyurea might affect fertility are not fully elucidated, it’s crucial to acknowledge that current research does not provide conclusive evidence of a direct and irreversible link between hydroxyurea and infertility.

However, until another therapeutic is identified with similar or better efficacy and safety, hydroxyurea remains one of the main managements for sickle cell disease-related vaso-occlusive crises.

**Figure 1:**
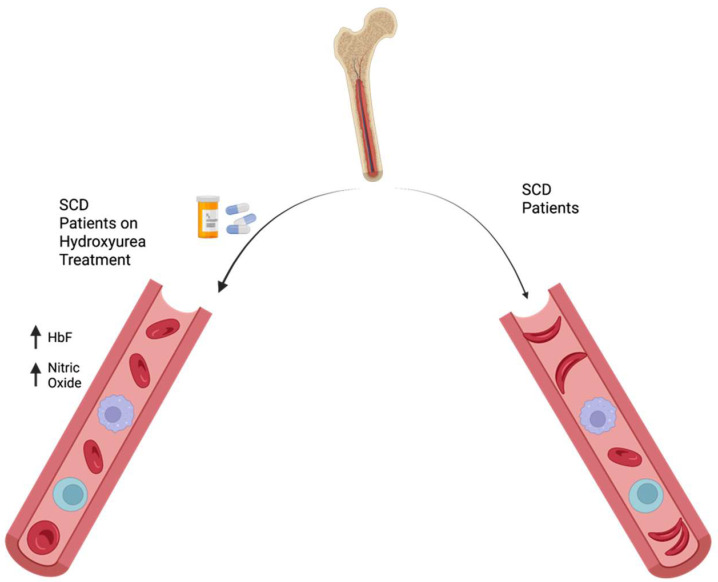
Schematic diagram illustrating two modes of action of hydroxyurea: (1) hydroxyurea elevates HbF levels in red blood cells and (2) increases the bioavailability of NO, inhibiting vasoconstriction and vaso-occlusion.

The action of hydroxyurea involves several mechanisms, some of which are still being investigated [7]. One action of hydroxyurea is to elevate fetal hemoglobin (HbF) levels in RBCs (Figure 1) [8]. HbF, a type of hemoglobin present in the blood of fetuses and newborns, prevents formation of the sickle cells that cause sickle cell anemia. One of the most important mechanisms of action is the inhibition of ribonucleotide reductase (RR), an enzyme involved in transforming ribonucleosides into deoxyribonucleosides that serve as building blocks for DNA synthesis [9,10]. Hydroxyurea is a potent RR inhibitor that reduces intracellular deoxynucleotide triphosphate pools and acts as an S-phase-specific agent, leading to the inhibition of DNA synthesis and eventual cellular cytotoxicity [11].

Hydroxyurea directly inhibits the RR M2 subunit, but spontaneous regeneration of the active enzyme occurs when hydroxyurea is removed. This results in predictably transient effects of hydroxyurea on RR in vivo, driven by the rapid absorption, metabolism, and excretion of hydroxyurea in mammalian systems. With once-daily dosing in sickle cell anemia, hydroxyurea causes intermittent cytotoxic suppression of erythroid progenitors and cell stress signaling. This then affects erythropoiesis kinetics and physiology, leading to the recruitment of erythroid progenitors with increased HbF levels. Additional mechanisms of action for HbF induction have been proposed for hydroxyurea, including free radical formation, iron chelation, activation of soluble guanylyl cyclase, and direct nitric oxide (NO) production [10,11,12].

The precise mechanism through which hydroxyurea stimulates HbF productions remains unknown; however, by elevating HbF levels in RBCs, hydroxyurea helps reduce the number of sickle cells and prevents formation of new sickle cells [13]. Consequently, hydroxyurea reduces the frequency and severity of vaso-occlusive crises and other complications associated with sickle cell disease [8,13].

Increased bioavailability of nitric oxide (NO) (Figure 1) has also been associated with hydroxyurea treatment [14,15]. NO, a molecule that plays an important role in regulating blood flow and preventing blood clot formation, stimulates production of cGMP, a chemical that causes blood vessels to relax and widen. In sickle cell patients, hemolysis of RBCs leads to reduced NO levels due to consumption of NO by free hemoglobin. By increasing NO production, hydroxyurea helps restore NO bioavailability and reduce the risk of vasoconstriction and blood clots [14,15]. Hydroxyurea has been shown to increase NO production through the enhanced release of endothelial nitric oxide synthase [16].

Another mechanism of action of hydroxyurea is reduction of the number of neutrophils, monocytes, and reticulocytes in the blood [3,13]. These cells play important roles in the development of inflammation and the formation of blood clots; therefore, by lowering the number of these white blood cells (WBCs), hydroxyurea helps to reduce the risk of inflammation and the formation of blood clots. Hydroxyurea inhibits ribonucleotide reductase, decreasing the levels of deoxyribonucleotide triphosphates and arresting proliferating cells in the S-phase. Through this mechanism, hydroxyurea may help reduce WBC counts [7,17].

Other mechanisms of hydroxyurea action may include increasing RBC size and volume and decreasing the phosphatidylserine density of RBCs [18,19]. Overall, hydroxyurea is effective for treating sickle cell anemia and related complications; however, some mechanisms of action require further investigation. The main actions of hydroxyurea are elevating HbF levels in RBCs, lowering the number of neutrophils, monocytes, and reticulocytes, and increasing NO bioavailability [2,20].

### 2.2. Blood Transfusions

Blood transfusions are important interventions for reducing morbidity and mortality in SCD patients, with more than 90% of SCD adults receiving at least one blood transfusion in their lifetime [21]. Transfusions help treat or prevent the perioperative complications, acute chest syndrome, and acute anemia associated with SCD; however, blood transfusions can also cause side effects such as alloimmunization and iron overload. Even though various strategies are employed to help mitigate these side effects, such as chelators for treating iron overload and antigen matching for preventing alloimmunization, these side effects persist [21,22].

## 3. New Treatments

For more than two decades, hydroxyurea was the only FDA-approved drug for SCD treatment. However, since 2017, three new SCD drugs have been approved by the FDA: voxelotor, crizanlizumab, and L-glutamine [1]. These new therapeutic treatments and associated mechanisms of action are discussed below, along with the only curative therapy currently available—hematopoietic stem cell transplantation from bone marrow. By definition, stem cell transplantation is not a new treatment, as bone marrow transplants were first used to treat SCD in 1984; however, important advancements in gene therapy have recently shown promising results.

### 3.1. Voxelotor

**Figure 2:**
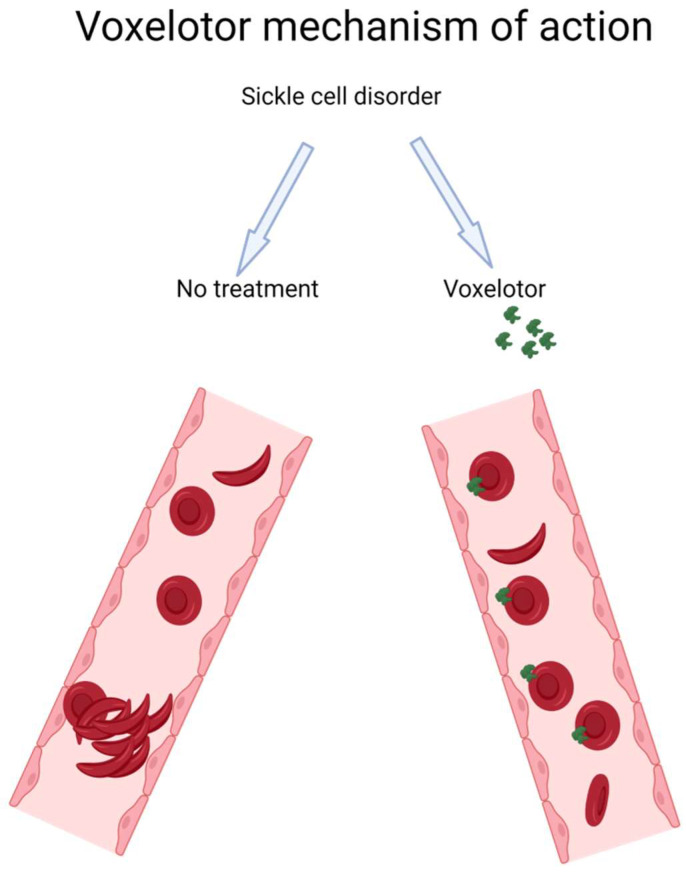
Schematic diagram of the mode of action of voxelotor, a drug that prevents sickled red blood cell formation in sickle cell disease. Voxelotor selectively binds to hemoglobin and increases its affinity for oxygen, keeping it in its oxygenated state and preventing sickling.

Voxelotor, approved by the FDA in 2019 for the treatment of sickle cell disease in adults and pediatric patients 12 years or older [23], belongs to a class of therapeutic agents called hemoglobin S polymerization inhibitors. These therapeutics work by changing the structure or function of abnormal hemoglobin, which prevents formation of sickled RBCs (Figure 2). Recent clinical trials investigating the efficacy and safety profile of voxelotor in SCD patients [24,25,26] showed increases in hemoglobin levels and decreases in laboratory markers of hemolysis, indicating that voxelotor reduces disease severity. The most common side effects of voxelotor were headache, diarrhea, nausea, rash, and abdominal pain.

Voxelotor acts by selectively and covalently binding to the N-terminal valine of the alpha-globin chain in hemoglobin molecules, modulating oxygen affinity [24]. This action prevents deoxygenation and polymerization of abnormal hemoglobin S, preventing the formation of sickled RBCs [24,25]. Other mechanisms of action have recently been attributed to the therapeutic effect of voxelotor in SCD. A study by Tarasev et al. explored the effect of GBT1118, a voxelotor analog, on hypoxia-induced lethal and sub-hemolytic RBC membrane damage [27,28]. Findings indicated that GBT1118 prevented hypoxia-induced membrane damage in sickled RBCs, indicating that voxelotor may also act through other mechanisms not related to hemoglobin–oxygen affinity.

### 3.2. Crizanlizumab

**Figure 3:**
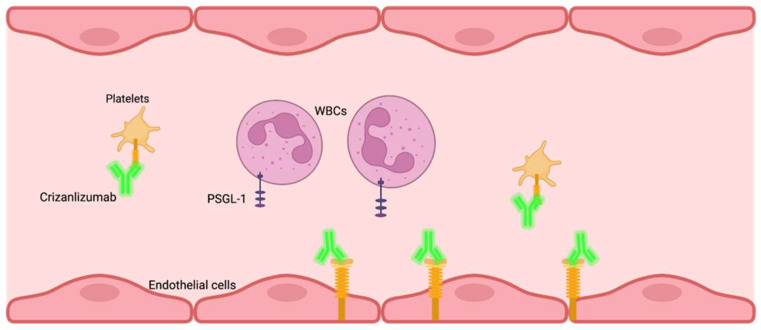
Schematic diagram of Crizanlizumab binding to P-selectin, a protein expressed on activated endothelial cells and platelets, inhibiting the adhesion of sickled red blood cells (RBCs) to the blood vessel walls. This reduces the formation of vaso-occlusive clusters of sickled RBCs, improving blood flow and reducing the frequency of vaso-occlusive events and hospital visits in sickle cell disease patients.

Crizanlizumab, approved by the FDA in 2019 to reduce the frequency of vaso-occlusive events in SCD adolescents and adults over 16 years of age [29], is an anti-P-selectin monoclonal antibody that binds P-selectin with high affinity blocking interactions between P-selectin and its ligands [30,31]. P-selectin, a protein found in Weibel-Palade bodies in endothelial cells and alpha granules in platelets [32], is expressed on the surface of platelets during activation. P-selectin glycoprotein ligand-1 (PSGL-1) is found on RBCs and WBCs [33], and when platelets are activated, the binding of PSGL-1 and P-selectin on platelets and endothelial cells leads to adhesion of sickled RBCs, WBCs, and platelets on the vascular endothelium, resulting in obstruction and hypoxia [22,23]. Crizanlizumab is administered intravenously and blocks interactions between P-selectin and sickled RBCs and WBCs, maintaining microvascular flow dynamics (Figure 3). Crizanlizumab is effective and safe for reducing the number of vaso-occlusive events and hospital visits, improving the quality of life for SCD patients [21,34,35].

However, it is noteworthy to mention that the European Medicines Agency (EMA) recently recommended revoking the marketing authorization for Crizanlizumab due to a reevaluation of its benefits and risks [36]. The decision was based on the assessment that the benefits of the medicine did not outweigh the potential risks associated with its use.

This recent development underscores the evolving nature of therapeutic options for sickle cell disease and the need for rigorous evaluation of both efficacy and safety profiles.

### 3.3. L-Glutamine

L-glutamine is an amino acid required for synthesis of nicotinamide adenine dinucleotide (NAD^+^) and its reduced form (NADH). In RBCs, NAD^+^ is an important antioxidant; therefore, L-glutamine is administered to SCD patients to improve the overall redox status of RBCs, reducing sickling, hemolysis, and vaso-occlusive crises. However, in the phase III trial used for FDA approval, no differences were observed in hemoglobin or hematocrit levels or reticulocyte counts in L-glutamine-treated SCD patients. These findings suggest that the benefits of L-glutamine may not be involved in reducing oxidative stress in RBCs [28,37].

### 3.4. Stem Cell Transplantation

Allogeneic hematopoietic stem cell transplantation (HCT) is not, by definition, a ‘new’ therapy, as it was first reported for SCD treatment in 1984; however, HCT consists of various methods that are continuously being improved [38].

HCT involves the administration of a healthy donor’s hematopoietic stem cells to modify SCD patient genotypes and remains the only curative treatment available for SCD [39]. Indications for HCT include stroke, recurrent hospitalizations or exchange transfusions for acute chest syndrome, and recurrent vaso-occlusive pain crises (≥3 episodes per year) [39,40,41,42]. However, HCT can result in graft rejection and cause graft versus host disease (GVHD)—the main cause of transplant-related mortality [40]. Due to associated risks, HCT use has been low and traditionally reserved for patients at risk of severe SCD-related complications [39,41,43]; however, advances in identifying human leukocyte antigen (HLA)-matched and haploidentical donors are improving HCT outcomes.

#### 3.4.1. HCT with HLA-Matched Donors

HLA-matching is used to match related or unrelated healthy donors with SCD patients for HCT. Excellent outcomes have been observed from HCT using HLA-identical matched sibling donors (MSD). Recent clinical trials have associated HCT in SCD patients with good outcomes, resulting in overall survival and event-free survival rates of 90% and approximately 85%, respectively [41]. Similarly, a study of 1000 SCD patients who received HLA-identical MSD HCT between 1986 and 2013 showed 93% survival [43]. However, few SCD patients have HLA-MSDs, so development of matched unrelated donor (MUD) programs is ongoing for HCT. Studies have reported high overall survival after MUD, but rejection rates and GVHD prevalence are high [44]. For example, an analysis of MUD HCT in seventy-one patients between 2005 and 2017 revealed an average three-year survival rate of 88%, with a 23% prevalence of three-year chronic GVHD [45]. This study also highlighted the importance of using 10/10 HLA-matched donors to improve survival, and, importantly, GVHD-free and relapse-free survival. However, identifying suitable HLA-matched donors and socio-economic factors still make HCT unavailable for many patients [41,43,45,46,47].

#### 3.4.2. HCT with Haploidentical Donors

Haploidentical HCT (haplo-HCT) is available to nearly all patients but has been traditionally associated with poor patient outcomes due to high incidences of graft failure and GVHD. However, strategies to prevent or treat GVHD are being developed. For example, T-cell depletion has improved outcomes pre- and post-haplo-HCT [48]. Overall, the robust use of T-cell depletion pre- and post-transplant and more supportive care have improved haplo-HCT outcomes. In fact, recent reviews on haplo-HCT have shown that the safety of this strategy has significantly improved and that it should be considered as a curative option for severe SCD cases [49].

## 4. Future Treatments

Several therapeutic treatments for SCD are in different stages of clinical development, such as prasugrel and oral anti-coagulants [50]. Curative approaches for treating SCD are also emerging, including gene therapy.

### 4.1. Prasugrel

Prasugrel, an oral therapeutic that inhibits adenosine diphosphate (ADP)-mediated platelet activation and aggregation, has been investigated in clinical trials for reductions in vaso-occlusive pain. Early trials showed promising trends, with decreased platelet activation and a reduction in vaso-occlusive pain observed [51,52]. However, observations from a multicenter multinational phase 3 clinical trial involving 341 children with SCD showed no significant decreases in vaso-occlusive pain events [53].

### 4.2. Mitapivat

Mitapivat, an innovative oral compound and the first of its kind, acts as an activator of erythrocyte pyruvate kinase (PKR). Originally explored in patients with pyruvate kinase deficiency (PKD), Mitapivat exhibited noteworthy improvements in hemoglobin (Hb) concentrations for non-transfusion-dependent patients and alleviated transfusion burdens for those undergoing regular transfusions [54]. Its approval in 2022 for PKD treatment marked a significant milestone. Furthermore, Mitapivat’s potential extends beyond PKD to encompass other hereditary chronic conditions linked to hemolytic mechanisms of anemia, including sickle cell disease (SCD) and thalassemia [55].

In the context of SCD, Mitapivat has been shown to be effective in a number of clinical trials, which illustrated Mitapivat’s dual impact [54,55,56,57,58]. Notably, it not only elevated Hb concentrations but also restored the thermostability of PKR, thereby enhancing its activity. Furthermore, it reduced levels of 2,3-diphosphoglycerate (2,3-DPG) in sickle erythrocytes. This reduction in 2,3-DPG levels led to decreased hemoglobin polymerization, bolstered by an increased affinity of hemoglobin for oxygen. With such multifaceted effects, Mitapivat demonstrated its potential to address the underlying mechanisms driving sickle cell pathophysiology.

### 4.3. Gene Therapy

One promising approach for treating SCD is the use of gene therapy to correct the genetic defect that causes SCD. A recent study reported findings from a phase 1/2 clinical trial using gene therapy to treat SCD [59]. In the trial, a lentiviral vector was used to deliver normal hemoglobin genes to stem cells of 15 patients with severe SCD. Specifically, autologous CD34+ cells transduced with a lentiviral vector encoding the human beta-globin gene were administered to patients through a single infusion. Results showed that the therapy was safe and well-tolerated, with no serious adverse events reported. Notably, the therapy increased production of normal hemoglobin and reduced the number of sickled RBCs [59]. This study provides promising evidence for the use of gene therapy as a treatment for SCD, although the study was small, and more research is needed to confirm the safety and efficacy of this potentially curative approach.

#### 4.3.1. Advanced Gene Editing Techniques in SCD Therapy

In addition to traditional gene therapy approaches, recent breakthroughs in genetic engineering have introduced innovative methods for treating sickle cell disease (SCD). Notably, gene editing techniques such as base editing and prime editing have garnered significant attention for their potential to correct the underlying genetic defects responsible for SCD.

##### Base Editing: Precision Redefined

Base editing represents a remarkable advancement that enables targeted modifications of specific DNA sequences without introducing double-strand breaks [60]. Through adenine base editors, researchers have achieved significant success in converting disease-causing alleles to non-pathogenic variants. Notably, these techniques have led to substantial improvements in hemoglobin production and reduction in sickling-related complications in preclinical models. Base editing’s precision and efficiency offer a promising alternative to conventional gene therapy methods [61,62].

A recent study by Zeng et al. demonstrated the feasibility of producing therapeutic levels of base edits in multilineage-repopulating and self-renewing human HSCs [62]. Base editing can potentially offer a high purity gene-corrected product compared to nuclease-based editing. Base editors directly introduce base changes without inducing DSBs, bypassing low-efficiency HDR as well as DSB-induced unwanted indels and off-target effects [63,64]. The A3A (N57Q)-BE3 base editor was delivered as an RNP targeting the BCL11A erythroid enhancer in SCD HSPCs [65]. This base editor targets cytosine within the base editing window to disrupt the GATA1 motif. Two cycles of electroporation increased the therapeutic base editing rate, but this also resulted in decreased viability and engraftment potential. Biallelic single base edits at the BCL11A enhancer within the GATA1 motif led to potent HbF induction similar to nuclease editing. Following transplantation into NBSGW mice, the base editing frequencies were reduced in engrafted HSCs compared to input HSPCs [63]. Base-edited cells showed multilineage reconstitution with similar base editing frequencies in each lineage. There was also erythroid lineage-specific BCL11A knockdown from erythroid enhancer disruption. For base editing, both gRNA-dependent and independent off-target editing need to be investigated [60,62,63,64,65]. Although off-target base editing can be minimized by reducing exposure to RNP and by utilizing the base editor with an attenuated cytosine deaminase domain, comprehensive off-target analysis needs to be performed before clinical implementation of base editing.

While promising, base editing for SCD treatment is not without challenges. The efficiency of this approach has not been tested in engrafting HSCs, and potential off-target effects and long-term consequences need to be thoroughly investigated [63,66,67]. Off-target base editing events, though minimized, still pose a concern that requires careful scrutiny and validation. The safety and durability of base editing-induced changes in the HBG promoter and subsequent HbF induction need to be demonstrated over time, especially in the context of HSC transplantation and long-term engraftments [63]. As with other gene editing strategies, the development of reliable monitoring methods to assess off-target effects and long-term safety will be crucial for advancing base editing as a viable therapeutic approach for SCD [66,67].

##### Prime Editing: Direct Correction at the Molecular Level

The realm of gene therapy for monogenic hematopoietic diseases, including sickle cell disease (SCD), has shown promise, yet hurdles such as cytotoxicity and mutagenicity linked to gene therapy vector delivery into patient hematopoietic stem cells (HSCs) continue to challenge clinical applications [68,69]. Recent instances of insertional oncogenesis highlight the potential consequences of unchecked gene expression driven by foreign promoters used in ex vivo gene therapy [69]. Additionally, safety concerns have arisen from ablative preconditioning regimens during HSC transplant.

An alternative avenue emerges with gene correction through CRISPR/Cas9 nuclease, which induces double-strand DNA breaks at mutated alleles for homology-directed repair. While addressing the disease-causing allele, CRISPR/Cas9 nuclease-based methods may raise HSC engraftment issues due to the DNA damage response elicited by both CRISPR/Cas9 and adeno-associated virus vectors [68,69].

To tackle these challenges, Li et al. introduce an innovative approach that could revolutionize gene therapy for SCD [68]. This method employs prime editing, a precise strategy that utilizes a catalytically impaired Cas9 nickase. Prime editing stands out by incorporating a reverse-transcribed RNA sequence within the target-specific prime editing guide RNA. This mechanism enables targeted DNA repair while significantly limiting the generation of unintended insertions and deletions at the target site, substantially enhancing safety [68,69].

Unlike CRISPR/Cas9 nuclease-based approaches that require coadministration of DNA templates through vectors, prime editing delivers the repair template directly through the primer-editing guide RNA [69,70]. This streamlined delivery reduces the risk of insertional mutagenesis and simplifies the therapeutic procedure.

In a groundbreaking study, Li et al. demonstrate successful beta-globin gene correction in mouse HSCs through intravenous administration of a prime editing vector [68]. This achievement is further highlighted by the maintenance of therapeutic levels of correction in secondary transplants, showcasing the successful editing of long-term repopulating HSCs.

However, it’s essential to acknowledge that attaining therapeutic levels necessitated in vivo selection with an alkylating agent to enhance the presence of a drug-resistance gene within the prime editing vector. Additionally, the authors exhibit the successful correction of human sickle cell disease patient HSCs ex vivo, with minimal unwanted insertions and deletions, along with no evidence of off-target effects [68].

Li et al.’s pioneering work introduces prime editing as a safer and more precise alternative for in vivo gene correction [68]. By addressing safety concerns associated with mutagenesis and preserving hematopoietic engraftment, prime editing offers a promising path forward in enhancing gene therapy’s effectiveness for SCD treatment. As research continues to refine this technique, the prospects of safer and more durable gene therapy for SCD patients become increasingly tangible.

##### Challenges and Future Directions

While base editing and prime editing show immense promise, challenges remain, including validation, optimization, and potential off-target effects [63,66,67]. Further research is needed to ensure the safety and long-term efficacy of these techniques in clinical settings. Additionally, considerations about delivery methods and ethical implications are vital for the successful translation of these technologies into viable treatments for SCD.

Although gene editing offers the potential for long-term benefits, the initial costs associated with research, development, and clinical implementation can be substantial. Moreover, the costs of ensuring safety, monitoring off-target effects, and conducting long-term follow-ups contribute to the overall financial burden.

## 5. Conclusions

In conclusion, there are many promising treatments for SCD that have been recently approved or are in development. Conventional treatments, such as hydroxyurea, have been used for decades and are effective for treating SCD-related painful episodes. However, hydroxyurea can produce a range of side effects, and the precise mechanisms of action for hydroxyurea are still under investigation. Recently the FDA-approved treatments, voxelotor, crizanlizumab, and L-glutamine, have also been proved effective for reducing the frequency and severity of vaso-occlusive crises and improving overall quality of life for SCD patients, providing additional treatment options for SCD. For curative treatments, stem cell transplantation remains the only curative treatment option available for SCD, but its use is limited by risks and challenges and is still in development. However, gene therapy has also shown promising results recently, and even though more studies and clinical trials are needed to investigate the efficacy and safety of this treatment option, this approach provides hope as a future curative treatment option.

## Data Availability

Not applicable.
